# Pilot investigation of circulating angiogenic and inflammatory biomarkers associated with vascular malformations

**DOI:** 10.1186/s13023-021-02009-7

**Published:** 2021-09-03

**Authors:** Sarah E. Wetzel-Strong, Shantel Weinsheimer, Jeffrey Nelson, Ludmila Pawlikowska, Dewi Clark, Mark D. Starr, Yingmiao Liu, Helen Kim, Marie E. Faughnan, Andrew B. Nixon, Douglas A. Marchuk

**Affiliations:** 1 Department of Molecular Genetics and Microbiology, Duke University Medical Center, 265 CARL Bldg., Box #3175 DUMC, Durham, NC 27710 USA; 2 Department of Anesthesia and Perioperative Care, University of California, San Francisco, San Francisco, CA USA; 3 Toronto HHT Centre, St. Michael’s Hospital and the Li Ka Shing Knowledge Institute, Toronto, Canada; 4 Department of Medicine, Duke Cancer Institute, Duke University Medical Center, Durham, NC USA; 5 Division of Respirology, Department of Medicine, University of Toronto, Toronto, Canada

**Keywords:** Vascular malformations, Biomarkers, HHT, CCM

## Abstract

**Background:**

Vascular malformations in the central nervous system are difficult to monitor and treat due to their inaccessible location. Angiogenic and inflammatory proteins are secreted into the bloodstream and may serve as useful biomarkers for identifying patients at risk for complications or with certain disease phenotypes.

**Methods:**

A validated multiplex protein array consisting of 26 angiogenic and inflammatory biomarkers (Angiome) was assessed in plasma isolated from healthy controls and patients with either sporadic brain arteriovenous malformation (BAVM), familial cerebral cavernous malformation (CCM), or hereditary hemorrhagic telangiectasia (HHT). These samples were obtained from archives of ongoing research studies at the University of California San Francisco and through prospective collection at the Toronto HHT Centre at St. Michael’s Hospital.

**Results:**

We compared circulating biomarker levels from each patient group to healthy controls and analyzed each pairwise combination of patient groups for differences in biomarker levels. Additionally, we analyzed the HHT samples to determine the association between biomarker levels and the following HHT-specific phenotypes, BAVM, pulmonary arteriovenous malformation (PAVM), liver vascular malformation (LVM), and gastrointestinal (GI) bleeding. Compared to controls, levels of SDF1 were significantly elevated in HHT patients (Proportional Increase [PI] = 1.87, *p* < 0.001, q = 0.011). Levels of sENG were significantly reduced in HHT patients compared to controls (PI = 0.56, *p* < 0.001, q < 0.001), reflecting the prevalence of HHT1 patients in this cohort. Levels of IL6 (PI = 3.22, *p* < 0.001, q < 0.001) and sTGFβR3 (PI = 0.70, *p* = 0.001, q < 0.029) differed significantly in CCM patients compared to controls. Compared to controls, ten of the biomarkers were significantly different in sporadic BAVM patients (q-values < 0.05). Among the pairwise combinations of patient groups, a significant elevation was observed in TGFβ1 in CCM patients compared to sporadic BAVM patients (PI = 2.30, *p* < 0.001, q = 0.034). When examining the association of circulating biomarker levels with HHT-specific phenotypes, four markers were significantly lower in HHT patients with BAVM (q-values < 0.05), and four markers were significantly higher in patients with LVM (q-values < 0.05).

**Conclusions:**

This pilot study suggests that the profile of circulating angiogenic and inflammatory biomarkers may be unique to each type of vascular malformation. Furthermore, this study indicates that circulating biomarkers may be useful for assessing phenotypic traits of vascular malformations.

**Supplementary Information:**

The online version contains supplementary material available at 10.1186/s13023-021-02009-7.

## Background

The Brain Vascular Malformation Consortium (BVMC) is a collaborative group of investigators dedicated to improving the care of patients with familial cerebral cavernous malformation (CCM), Sturge–Weber Syndrome (SWS), and hereditary hemorrhagic telangiectasia (HHT) [1]. Due to the presence of vascular malformations in the central nervous system, these patients may experience a range of debilitating and/or life-threatening symptoms including seizures, headache, and increased risk of cerebral hemorrhage. There is a lack of effective and targeted therapies for these patients and quality of life suffers as a result. Furthermore, these vascular malformations are difficult to monitor due to a reliance on imaging and the inability to biopsy the vascular malformations, while the localization of these lesions in the central nervous system complicates treatment. Currently it is challenging to predict which patients are at risk of worse outcomes. Therefore, it would be valuable to identify biomarkers from a non-invasive tissue source, such as blood, that are associated with disease phenotypes or outcomes.

Circulating biomarkers (or the liquid biopsy) have become an area of intense research interest over the past three decades. The cancer realm has extensively studied circulating biomarkers to examine correlations with clinical outcomes after drug treatment [2], to identify biomarkers that predict a patient’s response to a particular treatment [3], and to monitor disease progression or recurrence. One hallmark of cancer progression is the association of localized increases in angiogenic signals, promoting the development of new vasculature necessary to feed the growing tumor [4]. Secondly, numerous studies have demonstrated that tumors secrete various cytokines to recruit inflammatory cells and the resulting inflammation is involved in tumor progression [4]. Based on the angiogenic nature of many solid tumors and the rapid expansion of anti-angiogenic agents being used in the clinic, the Nixon lab developed a multiplex protein array that interrogates many key circulating biomarkers simultaneously. This biomarker panel, termed the Angiome, has been approved by the National Cancer Institute and the assay validated across numerous clinical studies [2, 5, 6].

Similar to cancer, angiogenic and inflammatory processes are dysregulated in vascular malformation diseases [7, 8]. In recent years, there has been increased interest in identifying circulating biomarkers associated with disease phenotypes in vascular malformation diseases. For example, a recent study used computational models to find associations between circulating biomarker levels and bleeding or hemorrhagic expansion of lesions in patients with CCM [9–11], demonstrating the feasibility of linking circulating markers to disease traits in vascular malformation diseases. Other studies have investigated the utility of non-coding RNAs, including microRNAs, as biomarkers for disease-associated phenotypes [12, 13]. One such study found that elevated levels of microRNA-210 in the circulation were associated with pulmonary arteriovenous malformation (PAVM) in patients with HHT [13], providing further support for our approach investigating biomarkers in the circulation for associations with disease phenotypes. Studies have also been conducted examining circulating biomarkers in HHT [14–16]; however, these studies have generally been limited to a few carefully selected markers involved in angiogenic processes. Therefore, additional studies, such as the pilot study reported here, are necessary to better distinguish disease-associated phenotypes and in the future, determine if there are circulating markers associated with measurable disease outcomes.

The goal of this pilot project was to determine if there are circulating angiogenic and/or inflammatory biomarkers that are associated with sporadic brain arteriovenous malformation (BAVM), familial CCM, and HHT. Although sporadic BAVM patients are not part of the BVMC, these samples were included as a comparison group to investigate whether the circulating markers in sporadically developing arteriovenous malformations (AVMs) differ from the markers in patients with inherited AVMs that occur in HHT. Many of the markers evaluated in our analysis have been previously shown to exhibit abnormal levels in each of these vascular malformation diseases [7, 8, 17], and thus we hypothesized that differences useful for characterizing each type of vascular malformation may be observed. This study also conducted preliminary analyses of whether circulating biomarkers may be associated with specific phenotypes in patients with HHT. Samples were unavailable for SWS at the time of this study and are therefore not included in this analysis.

## Results

### Patient demographics and HHT organ involvement

Summary statistics of patient and control characteristics by sample type are reported in Table 1. A total of 90 samples were included in the dataset (42 HHT, 20 controls, 18 CCM, and 10 sporadic BAVM). The mean patient age across all sample groups was 47.9 years old. Females made up 61% of the total samples. Of the 82 samples with race and ethnicity information available, 61 (74%) were non-Hispanic Caucasians and 21 (26%) were Hispanic. A summary of organ involvement among HHT patients is presented in Table 2. Among HHT patients, 20 (48%) had BAVM, 30 (71%) had pulmonary arteriovenous malformation (PAVM), 9 (21%) had gastrointestinal (GI) bleeding, and 3 of 37 with information (8%) had symptomatic liver vascular malformations (LVM). HHT organ involvement was defined as previously described [18].

**Table 1:** Patient characteristics

	HHT	CCM	Sporadic BAVM	Controls	Overall
Count	42	18	10	20	90
Patient age (y), mean ± SD	48.5 ± 13.9	52.1 ± 13.8	47.5 ± 17.2	43.2 ± 10.6	47.9 ± 13.7
Female, n (%)	25 (60%)	12 (67%)	4 (40%)	14 (70%)	55 (61%)
Caucasian (non-Hispanic), n/total (%)	31/36 (86%)	7/18 (39%)	6/10 (60%)	17/18 (94%)	61/82 (74%)

**Table 2:** HHT organ involvement

	HHT patients
Count	42
BAVM (y), n/total (%)	20/42 (48)
PAVM (y), n/total (%)	30/42 (71)
GI bleeding (y), n/total (%)	9/42 (21)
Symptomatic LVM (y), n/total (%)*	3/37 (8)

*Information about liver involvement was only available for 37 patients

### Biomarker levels in patients with vascular malformations compared to healthy controls

**Fig. 1:**
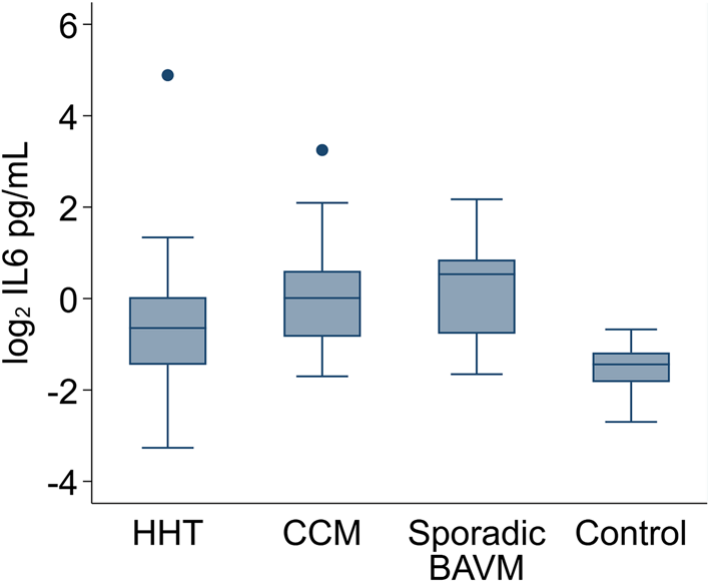
IL6 levels elevated in patients with vascular malformations compared to healthy controls. Log-transformed biomarker levels for patients with HHT, CCM, or sporadic BAVM and healthy controls were plotted

We compared circulating levels of each biomarker for each patient population (HHT, CCM, sporadic BAVM) to healthy controls. The regression results are presented in Table 3. When comparing HHT samples to healthy controls, stromal cell-derived factor 1 (SDF1) levels were significantly higher in HHT patients (Proportional Increase [PI] = 1.87, 95% confidence interval [CI] 1.34 to 2.62, *p* < 0.001, q = 0.011) and soluble endoglin (sENG) levels were significantly lower (PI = 0.56, 95% CI 0.45 to 0.69, *p* < 0.001, q < 0.001). When comparing CCM samples to healthy controls, interleukin 6 (IL6) levels were significantly higher in CCM patients (PI = 3.22, 95% CI 2.08 to 4.98, *p* < 0.001, q < 0.001, Fig. 1) and soluble transforming growth factor beta receptor 3 (sTGFβR3) levels were significantly lower (PI = 0.70, 95% CI 0.57 to 0.86, *p* = 0.001, q = 0.029). When sporadic BAVM samples were compared to healthy controls, several markers were found to differ significantly from controls, including glycoprotein 130 (GP130), IL6, soluble IL6 receptor (sIL6R), platelet-derived growth factor AA (PDGF-AA), transforming growth factor beta 1 (TGFβ1), sTGFβR3, tissue inhibitors of metalloproteinases 1 (TIMP1), thrombospondin 2 (TSP2), soluble vascular cell adhesion molecule 1 (sVCAM1), and soluble vascular endothelial growth factor receptor 1 (sVEGFR1) (see Table 3 for PI, CI, *p* values, and q-values). When we compared between the disease groups (i.e., HHT vs. CCM, HHT vs. sporadic BAVM, and CCM vs. sporadic BAVM), the only significant finding was an elevation of TGFβ1 in CCM samples compared to sporadic BAVM samples (PI = 2.30, 95% CI 1.45 to 3.63, *p* < 0.001, q = 0.034) (Additional file 1: Table S1).

**Table 3:** Linear regression of biomarker levels in patients with vascular malformations compared with controls

Marker	HHT versus controls				CCM versus controls				Sporadic BAVM versus controls			
	PI	95% CI	*p* value	q-value	PI	95% CI	*p* value	q-value	PI	95% CI	*p* value	q-value
ANG2	0.51	(0.24, 1.11)	0.089	1.000	0.52	(0.18, 1.49)	0.224	1	1.13	(0.30, 4.19)	0.857	1
BMP9	0.78	(0.28, 2.18)	0.629	1	0.94	(0.28, 3.14)	0.926	1	0.61	(0.11, 3.54)	0.581	1
sCD73	0.70	(0.41, 1.19)	0.188	1	0.67	(0.33, 1.35)	0.261	1	0.68	(0.29, 1.59)	0.373	1
sENG	**0.56**	**(0.45, 0.69)**	**< 0.001**	**< 0.001**	0.75	(0.59, 0.97)	0.027	0.620	0.85	(0.71, 1.03)	0.101	0.613
GP130	1.00	(0.89, 1.12)	0.996	1	0.93	(0.81, 1.05)	0.234	1	**0.79**	**(0.69, 0.91)**	**0.001**	**0.010**
HGF	2.42	(1.25, 4.66)	0.008	0.190	1.47	(0.84, 2.58)	0.182	1	3.56	(1.28, 9.88)	0.015	0.123
sICAM1	0.92	(0.80, 1.06)	0.263	1	0.98	(0.83, 1.17)	0.859	1	0.82	(0.69, 0.96)	0.017	0.127
IL6	1.65	(1.07, 2.55)	0.022	0.407	**3.22**	**(2.08, 4.98)**	**< 0.001**	**< 0.001**	**3.12**	**(1.97, 4.94)**	**< 0.001**	**< 0.001**
sIL6R	0.94	(0.79, 1.11)	0.465	1	0.88	(0.69, 1.11)	0.272	1	**0.62**	**(0.47, 0.83)**	**0.001**	**0.013**
OPN	0.99	(0.80, 1.24)	0.954	1	1.33	(1.01, 1.73)	0.039	0.706	0.84	(0.64, 1.12)	0.243	1
PDGF-AA	0.84	(0.53, 1.35)	0.479	1	0.84	(0.48, 1.47)	0.547	1	**0.42**	**(0.29, 0.61)**	**< 0.001**	**< 0.001**
PDGF-BB	1.17	(0.77, 1.76)	0.466	1	1.63	(0.95, 2.80)	0.075	1	0.69	(0.39, 1.23)	0.210	1
PIGF	1.26	(1.02, 1.57)	0.035	0.526	1.15	(0.84, 1.58)	0.395	1	1.40	(0.90, 2.19)	0.139	0.788
SDF1	**1.87**	**(1.34, 2.62)**	**< 0.001**	**0.011**	1.53	(0.91, 2.57)	0.106	1	1.62	(0.80, 3.28)	0.180	0.960
TGFβ1	0.98	(0.77, 1.24)	0.856	1	1.40	(1.11, 1.76)	0.005	0.140	**0.56**	**(0.49, 0.64)**	**< 0.001**	**< 0.001**
TGFβ2	0.95	(0.76, 1.19)	0.682	1	0.93	(0.72, 1.19)	0.547	1	0.76	(0.58, 0.99)	0.045	0.292
sTGFβR3	0.87	(0.73, 1.05)	0.142	1	**0.70**	**(0.57, 0.86)**	**0.001**	**0.029**	**0.59**	**(0.49, 0.73)**	**< 0.001**	**< 0.001**
TIMP1	0.96	(0.80, 1.16)	0.700	1	0.93	(0.76, 1.15)	0.518	1	**0.65**	**(0.53, 0.81)**	**< 0.001**	**0.002**
TSP2	0.85	(0.66, 1.10)	0.213	1	0.78	(0.58, 1.06)	0.109	1	**0.64**	**(0.48, 0.85)**	**0.002**	**0.018**
sVCAM1	0.97	(0.83, 1.14)	0.709	1	0.92	(0.74, 1.13)	0.423	1	**0.71**	**(0.57, 0.90)**	**0.004**	**0.041**
VEGF	0.84	(0.56, 1.26)	0.401	1	1.05	(0.69, 1.60)	0.822	1	0.83	(0.57, 1.23)	0.359	1
sVEGFR1	2.96	(1.44, 6.07)	0.003	0.095	1.15	(0.95, 1.39)	0.152	1	**3.60**	**(1.62, 8.01)**	**0.002**	**0.018**
sVEGFR2	0.97	(0.80, 1.18)	0.776	1	1.01	(0.80, 1.27)	0.938	1	0.71	(0.54, 0.95)	0.020	0.142
sVEGFR3	0.86	(0.73, 1.01)	0.065	0.845	0.98	(0.80, 1.21)	0.858	1	0.90	(0.69, 1.19)	0.471	1

*CI* confidence interval; *PI* proportional increase

Bold values indicate markers with a q-value less than 0.05. Sample sizes vary from 53 to 62 for HHT versus controls, 31 to 57 for CCM versus controls, and 25 to 30 for Sporadic BAVM versus controls

### Biomarker levels associated with disease phenotypes in HHT patients

Finally, we performed analyses using only HHT samples to assess the association of biomarker levels with disease phenotypes in HHT patients. The results for the HHT-specific analyses are presented in Table 4. We found that HHT patients with BAVM had lower levels of GP130 (PI = 0.78, 95% CI 0.68 to 0.89, *p* < 0.001, q = 0.017), soluble vascular endothelial growth factor receptor 3 (sVEGFR3) (PI = 0.75, 95% CI 0.63 to 0.88, *p* = 0.001, q = 0.017), soluble intracellular adhesion molecule 1 (sICAM1) (PI = 0.74, 95% CI 0.62 to 0.88, *p* = 0.001, q = 0.017), and TSP2 (PI = 0.59, 95% CI 0.44 to 0.80, *p* = 0.001, q = 0.017) compared to HHT patients without BAVM. We found that HHT patients with LVM had higher levels of TSP2 (PI = 3.39, 95% CI 2.08 to 5.53, *p* < 0.001, q < 0.001), sENG (PI = 2.07, 95% CI 1.35 to 3.18, *p* = 0.001, q = 0.028), GP130 (PI = 1.65, 95% CI 1.30 to 2.09, *p* < 0.001, q = 0.002), and TIMP1 (PI = 1.94, 95% CI 1.29 to 2.92, *p* = 0.002, q = 0.034) compared to patients without LVM documented. There were no biomarkers significantly associated with PAVM and GI bleeding among HHT patients.

**Table 4:** Linear regression of biomarkers levels associated with HHT-related phenotypes

Marker	BAVM				PAVM				LVM				GI bleeding			
	PI	95% CI	*p* value	q-value	PI	95% CI	*p* value	q-value	PI	95% CI	*p* value	q-value	PI	95% CI	*p* value	q-value
ANG2	1.18	(0.48, 2.91)	0.725	1	1.56	(0.59, 4.16)	0.371	1	0.43	(0.08, 2.30)	0.325	1	0.61	(0.20, 1.80)	0.368	1
BMP9	2.20	(0.66, 7.30)	0.197	1	0.64	(0.17, 2.35)	0.501	1	0.49	(0.05, 4.41)	0.522	1	1.87	(0.46, 7.54)	0.381	1
sCD73	1.11	(0.70, 1.76)	0.646	1	0.75	(0.45, 1.24)	0.256	1	0.79	(0.32, 1.99)	0.623	1	1.54	(0.89, 2.69)	0.125	1
sENG	0.76	(0.58, 1.00)	0.050	0.646	0.76	(0.56, 1.04)	0.084	1	**2.07**	**(1.35, 3.18)**	**0.001**	**0.028**	1.06	(0.75, 1.51)	0.725	1
GP130	**0.78**	**(0.68, 0.89)**	**< 0.001**	**0.017**	1.01	(0.85, 1.20)	0.930	1	**1.65**	**(1.30, 2.09)**	**< 0.001**	**0.002**	0.86	(0.72, 1.04)	0.112	1
HGF	1.26	(0.51, 3.13)	0.620	1	1.72	(0.64, 4.57)	0.280	1	0.24	(0.04, 1.35)	0.105	1	0.73	(0.24, 2.20)	0.575	1
sICAM1	**0.74**	**(0.62, 0.88)**	**0.001**	**0.017**	0.95	(0.77, 1.18)	0.648	1	1.50	(1.13, 1.98)	0.004	0.080	1.08	(0.85, 1.37)	0.516	1
IL6	1.13	(0.64, 1.98)	0.679	1	0.67	(0.35, 1.26)	0.209	1	0.64	(0.20, 2.05)	0.452	1	1.46	(0.73, 2.89)	0.283	1
sIL6R	0.88	(0.73, 1.08)	0.221	1	1.04	(0.84, 1.28)	0.750	1	1.27	(0.88, 1.83)	0.203	1	0.80	(0.64, 0.99)	0.043	1
OPN	1.05	(0.77, 1.43)	0.762	1	1.01	(0.73, 1.40)	0.962	1	0.75	(0.42, 1.34)	0.335	1	1.21	(0.86, 1.72)	0.278	1
PDGF-AA	0.55	(0.28, 1.07)	0.077	0.873	1.32	(0.63, 2.75)	0.458	1	3.83	(1.35, 10.87)	0.012	0.175	0.59	(0.27, 1.28)	0.181	1
PDGF-BB	0.93	(0.54, 1.60)	0.801	1	1.22	(0.68, 2.20)	0.503	1	0.93	(0.36, 2.42)	0.884	1	0.76	(0.39, 1.45)	0.404	1
PIGF	1.09	(0.86, 1.38)	0.480	1	1.26	(0.98, 1.61)	0.073	1	0.66	(0.42, 1.02)	0.062	0.805	1.05	(0.78, 1.40)	0.758	1
SDF1	0.93	(0.70, 1.24)	0.618	1	0.96	(0.69, 1.33)	0.786	1	0.83	(0.47, 1.45)	0.514	1	0.98	(0.68, 1.40)	0.902	1
TGFβ1	0.90	(0.63, 1.27)	0.546	1	0.96	(0.66, 1.40)	0.837	1	1.46	(0.77, 2.77)	0.241	1	1.12	(0.75, 1.67)	0.596	1
TGFβ2	0.98	(0.72, 1.34)	0.917	1	1.10	(0.79, 1.53)	0.574	1	1.20	(0.68, 2.11)	0.526	1	1.06	(0.74, 1.52)	0.756	1
sTGFβR3	0.86	(0.66, 1.11)	0.242	1	1.09	(0.82, 1.44)	0.560	1	1.12	(0.74, 1.69)	0.584	1	1.10	(0.82, 1.48)	0.536	1
TIMP1	0.74	(0.58, 0.94)	0.015	0.225	0.93	(0.70, 1.23)	0.607	1	**1.94**	**(1.29, 2.92)**	**0.002**	**0.034**	0.99	(0.73, 1.34)	0.923	1
TSP2	**0.59**	**(0.44, 0.80)**	**0.001**	**0.017**	1.04	(0.71, 1.52)	0.831	1	**3.39**	**(2.08, 5.53)**	**< 0.001**	**< 0.001**	0.83	(0.55, 1.24)	0.360	1
sVCAM1	0.87	(0.71, 1.06)	0.161	1	1.13	(0.91, 1.41)	0.265	1	1.29	(0.90, 1.86)	0.165	1	0.96	(0.76, 1.22)	0.749	1
VEGF	0.68	(0.38, 1.21)	0.192	1	0.97	(0.51, 1.85)	0.931	1	2.02	(0.64, 6.37)	0.228	1	1.42	(0.70, 2.88)	0.334	1
sVEGFR1	1.73	(0.65, 4.62)	0.271	1	1.23	(0.39, 3.82)	0.726	1	0.24	(0.03, 1.87)	0.173	1	0.56	(0.17, 1.87)	0.343	1
sVEGFR2	0.77	(0.63, 0.94)	0.011	0.199	1.06	(0.83, 1.36)	0.651	1	1.12	(0.72, 1.74)	0.603	1	0.93	(0.71, 1.22)	0.600	1
sVEGFR3	**0.75**	**(0.63, 0.88)**	**0.001**	**0.017**	0.99	(0.81, 1.22)	0.942	1	1.31	(0.94, 1.82)	0.110	1	0.96	(0.77, 1.20)	0.720	1

*CI* confidence interval; *PI* proportional increase

Bold values indicate markers with a q-value less than 0.05. Sample sizes vary from 33 to 42 for BAVM, PAVM, and GI bleeding, and 28 to 37 for LVM

## Discussion

In this pilot study, we characterized the plasma profile of 24 biomarkers that are known to drive angiogenesis and inflammation. We identified markers that were differentially expressed in patients with either sporadic BAVM, CCM, or HHT compared to healthy controls without known vascular anomalies. Among the different patient populations, we found that unique subsets of circulating markers existed across each group, with the exception of IL6, which was significantly higher in CCM and sporadic BAVM patients and trending higher in HHT patients. These data suggest that unique angiogenic and inflammatory processes may be involved in each disease, which may be useful for future monitoring of the patients as well as identifying the best course of treatment, at both the individual and disease level.

We identified several statistically significant associations that may be relevant to disease pathogenesis and phenotype presentation. When comparing the patient groups to healthy controls, we found a statistically significant elevation in IL6 levels in patients with CCM and sporadic BAVM and a non-significant elevation in patients with HHT (Fig. 1). These data suggest a shared role for IL6 in the pathology of vascular malformations. IL6 is a well-known multifunctional cytokine with reported roles in cardiovascular disease and endothelial dysfunction [19]. In recent years, there has been increased focus on the role of IL6 in the pathophysiology of vascular malformations. For example, polymorphisms in IL6 have been associated with hemorrhage in patients with sporadic BAVM [20]. More recently, another study found decreased levels of IL6 in patients with hemorrhagic activity of a lesion compared to an initial blood sample [9]. Since our study only assessed a single blood sample from each patient, we were unable to determine if these findings hold true in our patient population. Together, these findings indicate an important role for IL6 in the pathophysiology of vascular malformations, warranting further study.

When comparing CCM patients to healthy controls, we also found reduced levels of circulating TGFβR3, which has not been previously reported. Transforming growth factor beta (TGF-β) signaling is involved in regulating vascular development [21]. TGFβR3 belongs to one of the three types of TGF-β receptors, and it is widely expressed [21]. Although this receptor lacks kinase activity, and thus does not result in downstream signaling upon ligand binding, TGFβR3 is known to participate in ligand presentation to the other TGF-β receptors, especially to transforming growth factor beta receptor 2 (TGFβR2) [21]. Pertinent to this study, the soluble extracellular domain of TGFβR3 binds TGF-β in the circulation to antagonize signaling [21]. Increased TGF-β signaling has been implicated in the pathology of CCM [22, 23], specifically in the endothelial-mesenchymal transition in mature CCM lesions [23]. Together, these results indicate that further investigation into the role of TGF-β signaling in the context of CCM may be informative.

When comparing HHT samples to healthy controls, a statistically significant reduction in sENG was noted. However, when we restricted our analysis to the HHT samples for which genotyping information was available, we found that patients with HHT1 (mutations in *ENG*) had a 54% reduction in sENG compared to patients with HHT2 (mutations in *ACVRL1*) (PI = 0.54, 95% CI 0.42 to 0.69, *p* < 0.001, q < 0.001) (Additional file 2: Figure S1). Given that approximately 60% of the HHT cases with genetic information in our cohort have mutations in *ENG*, which matches the rates reported in other studies [24], these data indicate that the reduction observed in sENG is explained by the genetic basis of disease. This is further supported by a previous study demonstrating that HHT1 patients had significantly lower levels of sENG in the plasma compared to both HHT2 and healthy control groups [25]. These data also demonstrate that the HHT samples behave as expected with these ELISA-based arrays, strengthening the validity of our findings. Our current study also found significantly elevated levels of SDF1 only when comparing plasma samples from patients with HHT to healthy controls, which suggests the possibility of a unique role for this chemokine in the pathology of the vascular malformations in HHT. SDF1, also known as CXCL12, is a chemokine with a multitude of functions, including recruitment of bone marrow-derived endothelial progenitor cells (EPCs) [26] and involvement in patterning of the pulmonary arterial system [27]. With regards to vascular malformations, increased levels of SDF1 have been reported in the nidus of sporadic BAVMs, specifically in the endothelial and vascular smooth muscle cells of the malformation and is believed to contribute to the recruitment of EPCs to the AVM [28]. In the context of HHT, in vitro and animal model studies have suggested an interaction between SDF1 and ENG [29], including a role in regulating leukocyte transmigration across the endothelium [30] and in the regulation of SDF1 in the endothelium via BMP9 and ENG [31]. Therefore, further investigation into the role of SDF1 in the context of HHT, a form of familial AVMs, is warranted.

We found several statistically significant associations between the biomarkers assessed and HHT-associated BAVMs and LVMs. These associations may suggest different inflammatory and angiogenic processes influencing vascular malformations in specific organs. Among HHT patients with BAVMs compared to HHT patients without BAVM, we found significant reductions in sICAM1 and sVEGFR3, suggesting a unique role for these proteins in BAVMs among HHT patients. Elevated levels of sICAM1 have been reported in patients with subarachnoid hemorrhage [32, 33], and thus further investigation into the role of ICAM1 in HHT-associated BAVM may yield useful insights. VEGFR3 is one of three receptors for the vascular endothelial growth factors (VEGF) and is critically involved in the development of lymphatic vessels, a process known as lymphangiogenesis [34]. This receptor may also play a role in regulating blood vessel angiogenesis and permeability [34], suggesting that further investigation into the role of VEGFR3 in vascular malformations may be informative. We also found elevated levels of sENG and TIMP1 associated with LVMs among HHT patients. Since LVMs are more common in patients with mutations in *ACVRL1* [35, 36], the increase in sENG observed in this study is likely due to the genetic basis of disease, rather than a further increase above normal levels. TIMP1, one of four tissue inhibitors of metalloproteinases (TIMP), participates in the regulation of extracellular matrix proteins. Elevated levels of several TIMPs, including TIMP1, have been reported in the nidus of BAVMs from patients with sporadic BAVM [37], differing from the results published in this study. Further study is required to determine if this difference is due to the sample type assessed or clinical difference in the patient populations. In addition to the markers found to be associated with vascular involvement in a single organ, we found that GP130 and TSP2 had significantly different circulating levels in association with both BAVM and LVM in HHT patients. Interestingly, these markers were decreased in association with BAVMs, but increased in association with LVMs. These results may reflect different angiogenic and inflammatory environments to promote and maintain vascular malformations in these distinct organs or may reflect the underlying genetic basis of HHT since BAVMs are much more common in patients with HHT1, whereas LVMs are more common in patients with HHT2 [35, 36].

A limited number of studies have examined select circulating biomarkers, including angiogenic proteins and regulatory molecules such as microRNAs, in HHT patient populations [14–16, 38]. An older study reported reduced levels of TGFβ1 in the circulation of HHT1 patients [14], but this marker did not differ significantly in any of the comparisons assessed in our study. More recently, one study reported reduced levels of sVEGFR1 in the circulation of HHT2 patients compared to healthy controls [16]. Our current study failed to replicate these findings, which may be due to differences in anti-coagulant agents (EDTA vs. heparin) or due to the fact that our analysis did not stratify HHT patients based on genotype. Finally, one group found elevated levels of pentraxin 3 in plasma from HHT patients compared to healthy controls [15]. Interestingly, this marker was also more strongly associated with epistaxis-related scores than any other marker they assessed [15], supporting the hypothesis that there are indeed circulating biomarkers associated with disease-specific phenotypes. These studies, along with the data from our current study, highlight the need for further investigation into circulating factors that are associated with disease-specific phenotypes and events.

There are several caveats to this study that warrant discussion. First, this study of limited sample size is meant only to provide initial hypotheses that require validation in larger cohorts. Due to the limited sample size, we did not have sufficient power to conclusively show whether the underlying genetic cause of each disease (e.g., mutations in *ENG* vs. *ACVRL1* for HHT patients) influenced the profile of circulating biomarkers. Furthermore, this study was not sufficiently powered to detect small changes in circulating biomarker levels, unless these changes were very consistent among a disease group. Additional prospective, multi-site studies are required to expand the number of patients in each cohort to answer these important research questions. A second caveat of this study is that there were differences in the length of storage time between sample groups. Specifically, the sporadic BAVM and CCM samples were exclusively from bio-banked samples available through the BVMC, while approximately two-thirds of the HHT samples and all of the healthy control samples were collected prospectively from a single site. However, when we included the acquisition site as a correction factor in our analysis, as a proxy of storage time, the results did not appreciably change. Moving forward, future studies will be conducted with prospective sample collection. A third caveat is that this study was conducted using heparin plasma samples, which may skew some of the biomarker levels due to the presence of heparin-binding domains on some of the proteins, such as VEGF. However, this is a minor caveat since all samples analyzed were heparin plasma and this study was focused on characterizing in broad terms which markers were differentially present, rather than the absolute concentration of a particular marker in the blood.

## Conclusion

In conclusion, this pilot study found that the profile of circulating angiogenic and inflammatory biomarkers differs between patients with different types of vascular malformations, suggesting the importance of assessing individual conditions for relevant biomarkers. Our study also suggests that circulating biomarkers may represent a non-invasive method for assessing organ involvement in HHT patients, but this requires further validation. Future work should include patients with different types of vascular malformations and genetic etiologies, and address associations with prospective disease outcomes in patients with rare vascular malformations through longitudinal studies.

## Methods

### Samples and eligibility criteria

Heparin plasma samples were used to assess circulating angiogenic and inflammatory biomarkers levels. Frozen samples from sporadic BAVM and familial CCM patients were obtained from cases enrolled at the University of California San Francisco (UCSF) as part of ongoing research studies. HHT samples were obtained from either cases enrolled at UCSF as part of ongoing research studies (N = 26 patients) or were collected prospectively at the Toronto HHT Centre at St. Michael’s Hospital (N = 16 patients), after obtaining informed consent. Healthy control samples were prospectively collected at the Toronto HHT Centre at St. Michael’s Hospital, after obtaining informed consent.

All sporadic BAVM cases with confirmed diagnosis of shunting on conventional angiography who were seen at UCSF for evaluation or treatment were eligible to be enrolled into the parent study. Familial CCM cases were eligible to be enrolled at UCSF if they had a confirmed genetic mutation in one of the 3 known CCM genes or met 2 of 3 clinical criteria of CCM diagnosis, multiple brain lesions, and/or family history of CCM in first-degree relatives.

HHT cases were eligible to be enrolled if they had a confirmed genetic mutation in one of the 3 known HHT genes or met definite diagnosis of HHT by the clinical Curaçao criteria [39], which include: (1) recurrent and spontaneous epistaxis; (2) numerous telangiectasias present on the skin of the hands or in and around the nose and mouth; (3) vascular malformations, specifically AVMs and telangiectasias, affecting internal organs such as the brain, liver, and gastrointestinal tract; and (4) a family history of HHT. Cases with a history of severe anemia (hemoglobin [HB] < 80 g/L) in the last month or a blood transfusion within one month of the visit date were excluded from the study. HHT samples from both UCSF and St. Michael’s Hospital were included in this study.

Healthy controls were eligible for enrollment if they were 18 years of age or older. Individuals were excluded from the control arm using the following criteria: (1) a definite, probable, or likely HHT diagnosis; (2) a history of severe anemia (HB < 80 g/L) in the last month; and (3) a blood transfusion within one month of the visit date.

### Determination of circulating biomarker levels

Circulating biomarker levels were assessed using a panel multiplexed ELISA, termed the Angiome, as previously described [2, 5, 6]. Since the amount of plasma was limited for selected samples, not every biomarker was able to be assessed across all samples. We determined which markers to assay for each sample by assessing the volume requirements for each assay and selected the combination of markers that yielded the greatest number of biomarker data points for each sample. Of the 26 markers available as part of the Angiome panel, a total of 24 biomarkers were included in the analysis (Table 5). We were unable to run regression analyses on two of the markers, VEGF-C and VEGF-D, due to insufficient numbers of disease samples with data available.

**Table 5:** List of biomarkers assessed

ANG2	sICAM1	PIGF	TSP2	sVEGFR2
BMP9	IL6	SDF1	sVCAM1	sVEGFR3
sCD73	sIL6R	TGFβ1	VEGF	
sENG	OPN	TGFβ2	VEGF-C*	
GP130	PDGF-AA	sTGFβR3	VEGF-D*	
HGF	PDGF-BB	TIMP1	sVEGFR1	

*Data not presented due to insufficient sample size for linear regression

### Statistical methods

We used linear regression models to test whether individual biomarker levels were associated with individual phenotypes, while adjusting for age and sex. Phenotypes tested included patient type (e.g., HHT vs. control) and HHT-related organ involvement (e.g., HHT-related BAVM vs. non-BAVM). Models testing for HHT-related organ involvement included only HHT patients. Since many biomarkers were heavily right-skewed in distribution, we log-transformed all biomarker values to lessen the impact of outliers and better adhere to linear regression model assumptions. Two biomarkers, ANG2 and VEGF, had several readings below the limits of detection (LOD); for the purpose of data analysis, we set these readings to the midpoint between zero and the LOD before log-transformation. All results are presented as exponentiated coefficients, which we labeled as proportional increases (PI). A PI greater than one indicates an increase; for example, a PI of 1.2 is interpreted as 20% higher biomarker levels compared to the reference group. We additionally provide 95% confidence intervals (CI), *p* values, and q-values (q) derived from the Yekutieli method to correct for multiple testing. We focus on results with q-values less than 0.05. Stata version 15.1 was used to perform statistical analyses (College Station, TX: StataCorp LLC.).

## Supplementary Information

**Additional file 1: Table S1.** Linear regression of biomarker levels between vascular malformation diseases. CI = confidence interval; PI = proportional increase; Bold values indicate markers with a q-value less than 0.05. Sample sizes vary from 45 to 59 for HHT vs. CCM, 38 to 52 for HHT vs. Sporadic BAVM, and 17 to 27 for CCM vs. Sporadic BAVM.

**Additional file 2: Figure S1.** Soluble endoglin levels among HHT patients stratified by genotype. Log-transformed levels of sENG among HHT patients with genotype information were plotted. This analysis included data from 19 HHT1 patients (mutations in ENG), and 11 HHT2 patients (mutations in ACVRL1). Levels were significantly higher in HHT2 patients when adjusting for age and sex in a multivariable linear regression model (PI=1.85, 95% CI: 1.45 to 2.38, p<0.001).

## Data Availability

The datasets generated and analyzed during this current study will be made publicly available without patient identifiers.

## Abbreviations

AVM: Arteriovenous malformation
BAVM: Brain arteriovenous malformation
BVMC: Brain Vascular Malformation Consortium
CCM: Cerebral cavernous malformation
CI: Confidence interval
EPC: Endothelial progenitor cell
GI: Gastrointestinal
GP130: Glycoprotein 130
HB: Hemoglobin
HHT: Hereditary hemorrhagic telangiectasia
LOD: Limit of detection
LVM: Liver vascular malformation
PAVM: Pulmonary arteriovenous malformation
PDGFAA: Platelet-derived growth factor AA
PI: Proportional increase
SDF1: Stromal cell-derived factor 1
sENG: Soluble endoglin
sICAM1: Soluble intracellular adhesion molecule 1
IL6: Interleukin 6
sIL6R: Soluble interleukin 6 receptor
sTGFβR3: Soluble transforming growth factor beta receptor 3
sVCAM1: Soluble vascular cell adhesion molecule 1
sVEGFR1: Soluble vascular endothelial growth factor receptor 1
sVEGFR3: Soluble vascular endothelial growth factor receptor 3
SWS: Sturge–Weber Syndrome
TGFβ: Transforming growth factor beta
TGFβ1: Transforming growth factor beta 1
TIMP: Tissue inhibitors of metalloproteinases
TIMP1: Tissue inhibitors of metalloproteinases 1
TSP2: Thrombospondin 2
UCSF: University of California San Francisco
VEGF: Vascular endothelial growth factor
