# Supplementary figures and images for: Pilot investigation of circulating angiogenic and inflammatory biomarkers associated with vascular malformations
Source: Orphanet J Rare Dis. 2021 Sep 3;16:372. doi: 10.1186/s13023-021-02009-7 (PMC8414780; doi:10.1186/s13023-021-02009-7)

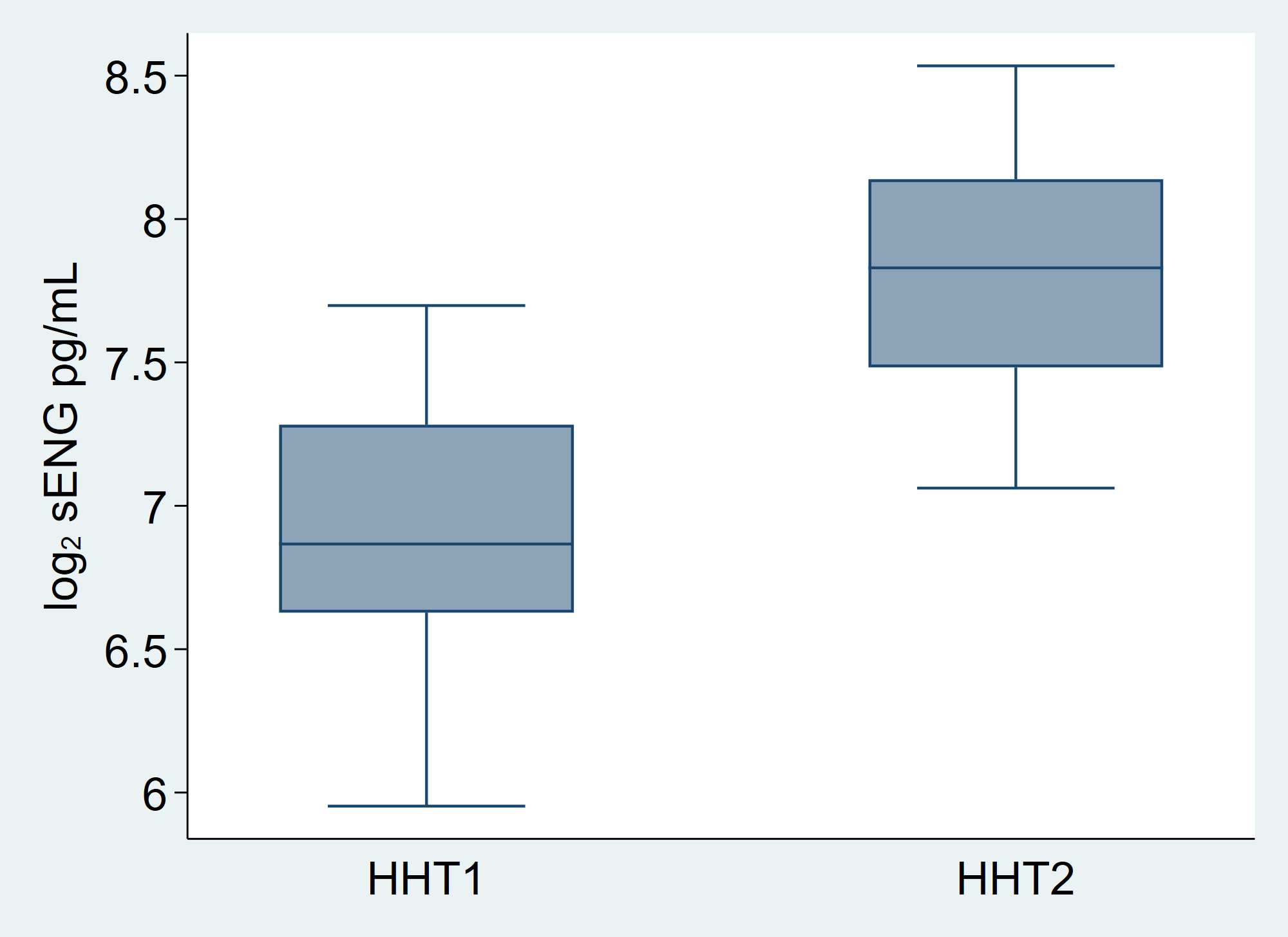

Supplement: Supplementary file 2 — Additional file 2: Figure S1. Soluble endoglin levels among HHT patients stratified by genotype. Log-transformed levels of sENG among HHT patients with genotype information were plotted. This analysis included data from 19 HHT1 patients (mutations in ENG), and 11 HHT2 patients (mutations in ACVRL1). Levels were significantly higher in HHT2 patients when adjusting for age and sex in a multivariable linear regression model (PI=1.85, 95% CI: 1.45 to 2.38, p<0.001). [file 13023_2021_2009_MOESM2_ESM.tiff]
